# Proceedings of the 2009 MidSouth Computational Biology and Bioinformatics Society (MCBIOS) Conference

**DOI:** 10.1186/1471-2105-10-S11-S1

**Published:** 2009-10-08

**Authors:** Jonathan D Wren, Yuriy Gusev, Raphael D Isokpehi, Daniel Berleant, Ulisses Braga-Neto, Dawn Wilkins, Susan Bridges

**Affiliations:** 1 Arthritis and Immunology Research Program, Oklahoma Medical Research Foundation; 825 N.E. 13th Street, Oklahoma City, OK 73104-5005, USA; 2 Department of Surgery, Health Sciences Center, The University of Oklahoma, Oklahoma City, OK 73104, USA; 3 College of Science, Engineering and Technology, Jackson State University, Jackson, MS, USA; 4 Department of Information Science, University of Arkansas at Little Rock, Little Rock, AR 72204, USA; 5 Department of Electrical and Computer Engineering, Texas A&M University, College Station, 77843-3128, USA; 6 Computer & Information Science Department, The University of Mississippi, University, MS 38677, USA; 7 Department of Computer Science and Engineering, Mississippi State University, Box 9637, Mississippi State, MS 39762, USA

## Introduction

MCBIOS 2009 was held February 20–21, 2009 at the Hunter Henry Center in Starkville, Mississippi on the Mississippi State University campus. The conference was hosted by the four research universities in Mississippi comprising the Mississippi Computational Biology Consortium – Jackson State University (JSU), Mississippi State University (MSU), the University of Mississippi (UM), and the University of Southern Mississippi (USM). Dr. Dawn Wilkins of UM and Dr. Susan Bridges of MSU served as conference co-chairs. Keynote speakers were Dr. Laura Elnitski, Head of the Genomic Functional Analysis Section at the National Human Genome Research Institute (NHGRI), Howard Cash, President and CEO of Gene Codes Corporation, and Dr. Cathy Wu, Director of the Protein Information Resource (PIR).

Dr. Raphael Isokpehi of JSU organized a panel discussion entitled Careers in Computational Biology and Bioinformatics: Perspective from Employers and Students. Panelists were Dr. Ed Perkins of the U.S. Army Engineering Research and Development Center, Robert Cottingham of Oak Ridge National Laboratory (ORNL), Dr. Doris M. Kupfer, Federal Aviation Administration, James C. Fuscoe, FDA National Center for Toxicological Research, and Cynthia Jeffries, student intern at ORNL. Over 40 student and faculty attendees also participated in the Speed Networking event organized by Dr. Isokpehi and featured in a recent *Science Careers *article [1]. Each person in the event spent 3 minutes talking to twenty other participants in the two hour session providing students an opportunity to interact with employers and with students from other universities in the region.

Dr. Bindu Nanduri and Dr. Andy Perkins, both of MSU managed the setup, judging, and scoring of 80 posters. Monetary awards were provided by Dr. Ed Perkins. Student poster award winners were: first place – Eric Morales of the University of New Orleans (UNO), second place Teresia Buza of MSU, third place – Prashanti Manda of MSU and honorable mention to Amanda Alba of the UNO, Surya Saha of MSU and Sudhir Chowbina of the Indiana University School of Informatics. Student winners for oral presentations were: first place – Enis Afgan of the University of Alabama at Birmingham, second place – Lipi R. Archarya of UNO, and third place – Anastasia Chueva of Mississippi Valley State University.

## Proceedings summary

Thirty-two papers from the 2009 conference were submitted to be considered for inclusion in this supplement, and of them a total of 20 were accepted (62.5% accept rate), making this year's Proceedings the most stringent to date. The number of submitted papers exceeded that of the 2008 MCBIOS Proceedings [2-20]. As in prior conferences, we strove to be inclusive yet rigorous in the peer-review process, so that the end result is both high quality and reflective of the work presented. Papers generally fell into five categories:

## Systems biology

A means of better understanding how systems work and interconnect as opposed to the traditional approach of isolating subcomponents and focusing on them is called Systems Biology and has become an increasingly active area of study in bioinformatics [21-34]. Andrey Ptitsyn offers a systems biology approach to the analysis of microarray data [35], partitioning gene expression space into a multi-dimensional taxonomy. Differences between "taxa" are then studied using biological pathway analysis software to associate gene expression patterns with specific phenotypes. For example, the application identifies a link between glycine metabolism aberrations and metabolic biomarkers for metastatic prostate cancer.

Gao *et al. *[36] describe an approach to better integrate data from disparate sources to reach better conclusions about biological response to stimulus than could be obtained from any of the component sources alone. Their study combined the Gene Ontology (GO) with microarray data using a bigraph data structure and associated algorithms.

Chowbina *et al. *address data integration from a different perspective[37], describing a technical resource for regulatory, signaling and biochemical reactions in human biomolecular pathways. Their resource, Human Pathway Database (HPD), integrates several existing resources to provide a web interface to access approximately one thousand pathways.

Zhang *et al. *present text empirics in the context of the PathBinder system [38]. Text empirics refers to properties of texts that are derived by examining the texts themselves. It differs from the machine learning approach in that derived properties are manually curated and presented as facts that system builders can use when they are designing their systems.

## OMICS

"OMICS" is a broad category, based on the fact that fields of study for biological entities often end with the suffix "omics" (e.g., transcriptomics, proteomics) [39-45]. Pendarvis *et al. *[46] describe the creation of a workflow system to analyze mass spectrometry replicate datasets to generate a list of identified proteins and expression changes and link them to biological knowledge. Because it is compliant with proteomic data submission guidelines, users can readily publish data to public proteomic data repositories.

Mete *et al. *[47] present a novel digital image analysis approach to the automated evaluation of angiogenesis in Liver Cancer. The method identifies regional, morphological and fractal features and helps measure micro-vessel density on digitized images of liver tumor sections. Their results show agreement between automated calculations and manual counting of micro-vessels.

## Microarray studies

Microarrays have long been a subject of bioinformatics analysis not only for better understanding transcription, but also as a model for large-scale analysis of genetic behavior [48-56]. Van Den Berg *et al. *compared gene annotation enrichment tools for functional modeling of agricultural microarray data [57], a category that is underrepresented among current tools. Chavan *et al. *describe Network Analysis Toolbox in R (NATbox), which provides a menu-driven GUI for modeling and analysis of functional relationships inferred from gene expression profiles. It is suited for interdisciplinary researchers and biologists with minimal programming experience who would like to use systems biology approaches without delving into the algorithmic aspects, and can be a useful demonstration/teaching tool.

Perkins and Langston [59] identify a means of effectively selecting thresholds for gene expression on microarrays by identifying "cliques" or subgroups of co-expressed genes. Roughly, the idea being that individual measurements are prone to error, but when a gene is seen co-expressed with other genes, then this reinforces the validity of the expression being biologically significant.

Li *et al. *[60] investigate inter- and intra-platform consistency of microarray measurements. Using pathway data as a guide for identifying biologically consistent results, the authors demonstrate that highly consistent biological information can be generated from different microarray platforms.

## Genomic analysis

T.J. Jankun-Kelly and colleagues present a method to visually explore conserved domains on Multiple Sequence Alignments [61], simultaneously communicating the relative similarity of proteins across species and the differences in how function is expressed via conserved domains. It quickly identifies conserved domains and allows both macro (sequence-long) and micro (small amino-acid neighborhood) views.

Xu *et al. *[62] report the results of an extensive comparative genomics analysis of plant lignin biosynthesis gene families. Lignin is a major component of plant cell walls and appears to be the major cause of cell wall recalcitrance in energy production. Their analysis confirms that, among the species analyzed, the first complete lignin biosynthesis pathway appeared in moss. The major expansion of biosynthesis families occurred after the divergence of monocots and dicots and duplicated genes had many different fates. They conclude that transgenic lignin modification strategies to bioenergy feedstock may only be successful between closely related species.

T. Buza *et al. *report a method of automated functional annotation of the chicken genome, improving both the breadth and quality of annotation of genes [63]. The method can facilitate functional annotation of other microarrays via an Array GO Mapper (AGOM) tool to help researchers quickly retrieve corresponding functional information.

Zhou *et al. *[64] develop CPTRA (Cross-Platform Transcriptome Analysis) to analyze Next Generation Sequencing based on Transcriptome profiling in species with limited genome information.

## Miscellaneous

Garcia-Reyero *et al. *[65] study gene expression in fathead minnows (*Pimephales promelas*) in well-characterized sites adjacent to sewage treatment plants and find short-term exposure to effluents were able to induce a site-specific gene expression pattern in the fathead minnow gonad and liver and to affect fish sexual behavior.

There are several other papers published in the supplement but, for space, are not summarized here: Chen and Johnson [66], Bright *et al. *[67], Zollanvari *et al. *[68] and Malone *et al. *[69].

## Future meetings

MCBIOS 2010 will be held in Jonesboro, Arkansas, and hosted by Arkansas State University and the Arkansas Biosciences Institute. Daniel Berleant of University of Arkansas at Little Rock is the 2009–2010 President of MCBIOS and will be chair of MCBIOS 2010. Ulisses Braga-Neto of Texas A&M University is the new President-elect of the society. See http://www.MCBIOS.org for information regarding MCBIOS and future meetings. MCBIOS is a regional affiliate of the International Society for Computational Biology http://www.ISCB.org.

## Competing interests

The authors declare that they have no competing interests.

## Authors' contributions

All authors served as co-editors for these proceedings, with JDW serving as Senior Editor. All authors helped write this editorial.

## References

[B1] Holmes L Perspective: Speed Networking for Scientists Science Careers 2009

[B2] Bottoms CA Xu D Wanted: unique names for unique atom positions. PDB-wide analysis of diastereotopic atom names of small molecules containing diphosphate BMC Bioinformatics 2008 9 Suppl 9 S16 10.1186/1471-2105-9-S9-S16 18793461 PMC2537567

[B3] Chae M Shmookler Reis RJ Thaden JJ An iterative block-shifting approach to retention time alignment that preserves the shape and area of gas chromatography-mass spectrometry peaks BMC Bioinformatics 2008 9 Suppl 9 S15 10.1186/1471-2105-9-S9-S15 18793460 PMC2537566

[B4] Churbanov A Winters-Hilt S Clustering ionic flow blockade toggles with a mixture of HMMs BMC Bioinformatics 2008 9 Suppl 9 S13 10.1186/1471-2105-9-S9-S13 18793458 PMC2537564

[B5] Dozmorov MG Kyker KD Hauser PJ Saban R Buethe DD Dozmorov I Centola MB Culkin DJ Hurst RE From microarray to biology: an integrated experimental, statistical and in silico analysis of how the extracellular matrix modulates the phenotype of cancer cells BMC Bioinformatics 2008 9 Suppl 9 S4 10.1186/1471-2105-9-S9-S4 18793468 PMC2537575

[B6] Frank RL Kandoth C Ercal F Validation of an NSP-based (negative selection pattern) gene family identification strategy BMC Bioinformatics 2008 9 Suppl 9 S2 10.1186/1471-2105-9-S9-S2 18793465 PMC2537573

[B7] Giles CB Wren JD Large-scale directional relationship extraction and resolution BMC Bioinformatics 2008 9 Suppl 9 S11 10.1186/1471-2105-9-S9-S11 18793456 PMC2537562

[B8] Hong H Su Z Ge W Shi L Perkins R Fang H Xu J Chen JJ Han T Kaput J Assessing batch effects of genotype calling algorithm BRLMM for the Affymetrix GeneChip Human Mapping 500 K array set using 270 HapMap samples BMC Bioinformatics 2008 9 Suppl 9 S17 10.1186/1471-2105-9-S9-S17 18793462 PMC2537568

[B9] Huan T Sivachenko AY Harrison SH Chen JY ProteoLens: a visual analytic tool for multi-scale database-driven biological network data mining BMC Bioinformatics 2008 9 Suppl 9 S5 10.1186/1471-2105-9-S9-S5 18793469 PMC2537576

[B10] Kulkarni V Errami M Barber R Garner HR Exhaustive prediction of disease susceptibility to coding base changes in the human genome BMC Bioinformatics 2008 9 Suppl 9 S3 10.1186/1471-2105-9-S9-S3 18793467 PMC2537574

[B11] Lee T Desai VG Velasco C Reis RJ Delongchamp RR Testing for treatment effects on gene ontology BMC Bioinformatics 2008 9 Suppl 9 S20 10.1186/1471-2105-9-S9-S20 18793466 PMC2537571

[B12] Mete M Tang F Xu X Yuruk N A structural approach for finding functional modules from large biological networks BMC Bioinformatics 2008 9 Suppl 9 S19 10.1186/1471-2105-9-S9-S19 18793464 PMC2537570

[B13] Ptitsyn A Comprehensive analysis of circadian periodic pattern in plant transcriptome BMC Bioinformatics 2008 9 Suppl 9 S18 10.1186/1471-2105-9-S9-S18 18793463 PMC2537569

[B14] Ptitsyn AA Weil MM Thamm DH Systems biology approach to identification of biomarkers for metastatic progression in cancer BMC Bioinformatics 2008 9 Suppl 9 S8 10.1186/1471-2105-9-S9-S8 18793472 PMC2537559

[B15] Quest D Dempsey K Shafiullah M Bastola D Ali H MTAP: the motif tool assessment platform BMC Bioinformatics 2008 9 Suppl 9 S6 10.1186/1471-2105-9-S9-S6 18793470 PMC2537577

[B16] Rawat A Seifert GJ Deng Y Novel implementation of conditional co-regulation by graph theory to derive co-expressed genes from microarray data BMC Bioinformatics 2008 9 Suppl 9 S7 10.1186/1471-2105-9-S9-S7 18793471 PMC2537558

[B17] Roux B Winters-Hilt S Hybrid MM/SVM structural sensors for stochastic sequential data BMC Bioinformatics 2008 9 Suppl 9 S12 10.1186/1471-2105-9-S9-S12 18793457 PMC2537563

[B18] Shi L Jones WD Jensen RV Harris SC Perkins RG Goodsaid FM Guo L Croner LJ Boysen C Fang H The balance of reproducibility, sensitivity, and specificity of lists of differentially expressed genes in microarray studies BMC Bioinformatics 2008 9 Suppl 9 S10 10.1186/1471-2105-9-S9-S10 18793455 PMC2537561

[B19] Su Z Hong H Fang H Shi L Perkins R Tong W Very Important Pool (VIP) genes – an application for microarray-based molecular signatures BMC Bioinformatics 2008 9 Suppl 9 S9 10.1186/1471-2105-9-S9-S9 18793473 PMC2537560

[B20] Zielinski JS Bouaynaya N Schonfeld D O'Neill W Time-dependent ARMA modeling of genomic sequences BMC Bioinformatics 2008 9 Suppl 9 S14 10.1186/1471-2105-9-S9-S14 18793459 PMC2537565

[B21] Centler F Kaleta C di Fenizio PS Dittrich P Computing chemical organizations in biological networks Bioinformatics 2008 24 1611 1618 10.1093/bioinformatics/btn228 18480100

[B22] Li Y Agarwal P Rajagopalan D A global pathway crosstalk network Bioinformatics 2008 24 1442 1447 10.1093/bioinformatics/btn200 18434343

[B23] Mazurie A Bonchev D Schwikowski B Buck GA Phylogenetic distances are encoded in networks of interacting pathways Bioinformatics 2008 24 2579 2585 10.1093/bioinformatics/btn503 18820265 PMC2579716

[B24] Vyshemirsky V Girolami M BioBayes: a software package for Bayesian inference in systems biology Bioinformatics 2008 24 1933 1934 10.1093/bioinformatics/btn338 18632751

[B25] Weidemann A Richter S Stein M Sahle S Gauges R Gabdoulline R Surovtsova I Semmelrock N Besson B Rojas I SYCAMORE – a systems biology computational analysis and modeling research environment Bioinformatics 2008 24 1463 1464 10.1093/bioinformatics/btn207 18463116

[B26] Xiong H Choe Y Structural systems identification of genetic regulatory networks Bioinformatics 2008 24 553 560 10.1093/bioinformatics/btm623 18175769

[B27] Suderman M Hallett M Tools for visually exploring biological networks Bioinformatics 2007 23 2651 2659 10.1093/bioinformatics/btm401 17720984

[B28] Saeys Y Inza I Larranaga P A review of feature selection techniques in bioinformatics Bioinformatics 2007 23 2507 2517 10.1093/bioinformatics/btm344 17720704

[B29] Hibbs MA Hess DC Myers CL Huttenhower C Li K Troyanskaya OG Exploring the functional landscape of gene expression: directed search of large microarray compendia Bioinformatics 2007 23 2692 2699 10.1093/bioinformatics/btm403 17724061

[B30] Avila-Campillo I Drew K Lin J Reiss DJ Bonneau R BioNetBuilder: automatic integration of biological networks Bioinformatics 2007 23 392 393 10.1093/bioinformatics/btl604 17138585

[B31] Schmidt H SBaddon: high performance simulation for the Systems Biology Toolbox for MATLAB Bioinformatics 2007 23 646 647 10.1093/bioinformatics/btl668 17237071

[B32] Schmidt H Drews G Vera J Wolkenhauer O SBML export interface for the systems biology toolbox for MATLAB Bioinformatics 2007 23 1297 1298 10.1093/bioinformatics/btm105 17384017

[B33] Shah AR Singhal M Klicker KR Stephan EG Wiley HS Waters KM Enabling high-throughput data management for systems biology: the Bioinformatics Resource Manager Bioinformatics 2007 23 906 909 10.1093/bioinformatics/btm031 17324940

[B34] Li P Oinn T Soiland S Kell DB Automated manipulation of systems biology models using libSBML within Taverna workflows Bioinformatics 2008 24 287 289 10.1093/bioinformatics/btm578 18056069

[B35] Ptitsyn A Computational analysis of gene expression space associated with metastatic cancer BMC Bioinformatics 2009 10 Suppl 11 S6 10.1186/1471-2105-10-S11-S6 19811690 PMC3226195

[B36] Gao C Dang X Chen Y Wilkins D Graph ranking for exploratory gene data analysis BMC Bioinformatics 2009 10 Suppl 11 S20 10.1186/1471-2105-10-S11-S19 PMC3226190 19811684

[B37] Chowbina SR Wu X Zhang F Li PM Pandy R Kasamsetty HN Chen JY HPD: An Online Integrated Human Pathway Database Enabling Systems Biology Studies BMC Bioinformatics 2009 10 Suppl 11 S5 10.1186/1471-2105-10-S11-S5 19811689 PMC3226194

[B38] Zhang L Berleant D Ding J Cao T Wurtele ES PathBinder – text empirics and automatic extraction of biomolecular interactions BMC Bioinformatics 2009 10 Suppl 11 S18 10.1186/1471-2105-10-S1-S18 19811683 PMC3226189

[B39] Toyoda T Mochizuki Y Player K Heida N Kobayashi N Sakaki Y OmicBrowse: a browser of multidimensional omics annotations Bioinformatics 2007 23 524 526 10.1093/bioinformatics/btl523 17077097

[B40] Polpitiya AD Qian WJ Jaitly N Petyuk VA Adkins JN Camp DG 2nd Anderson GA Smith RD DAnTE: a statistical tool for quantitative analysis of -omics data Bioinformatics 2008 24 1556 1558 10.1093/bioinformatics/btn217 18453552 PMC2692489

[B41] Xia T Dickerson JA OmicsViz: Cytoscape plug-in for visualizing omics data across species Bioinformatics 2008 24 2557 2558 10.1093/bioinformatics/btn473 18779234 PMC2572702

[B42] Gong L Owen RP Gor W Altman RB Klein TE PharmGKB: an integrated resource of pharmacogenomic data and knowledge Curr Protoc Bioinformatics 2008 Chapter 14 Unit 14 17 10.1002/0471250953.bi1407s23 PMC4153752 18819074

[B43] Kao HL Gunsalus KC Browsing multidimensional molecular networks with the generic network browser (N-Browse) Curr Protoc Bioinformatics 2008 Chapter 9 Unit 9 11 18819079 10.1002/0471250953.bi0911s23 PMC3217184

[B44] Martens L Jones P Cote R Using the Proteomics Identifications Database (PRIDE) Curr Protoc Bioinformatics 2008 Chapter 13 Unit 13 18 10.1002/0471250953.bi1308s21 18428683

[B45] Yeung N Cline MS Kuchinsky A Smoot ME Bader GD Exploring biological networks with Cytoscape software Curr Protoc Bioinformatics 2008 Chapter 8 Unit 8 13 18819078 10.1002/0471250953.bi0813s23

[B46] Pendarvis K Kumar R Burgess SC Nanduri B An automated proteomic data analysis workflow for mass spectrometry BMC Bioinformatics 2009 10 Suppl 9 S17 10.1186/1471-2105-10-S11-S17 19811682 PMC3226188

[B47] Mete M Hennings L Spencer HJ Topaloglu U Automatic Identification of Angiogenesis in Double Stained Images of Liver Tissue BMC Bioinformatics 2009 10 Suppl 11 S13 10.1186/1471-2105-10-S11-S13 19811678 PMC3226185

[B48] Burkart MF Wren JD Herschkowitz JI Perou CM Garner HR Clustering microarray-derived gene lists through implicit literature relationships Bioinformatics 2007 23 1995 2003 10.1093/bioinformatics/btm261 17537751

[B49] Hong F Breitling R A comparison of meta-analysis methods for detecting differentially expressed genes in microarray experiments Bioinformatics 2008 24 374 382 10.1093/bioinformatics/btm620 18204063

[B50] Li H Zhan M Unraveling transcriptional regulatory programs by integrative analysis of microarray and transcription factor binding data Bioinformatics 2008 24 1874 1880 10.1093/bioinformatics/btn332 18586698 PMC2519161

[B51] Yang D Li Y Xiao H Liu Q Zhang M Zhu J Ma W Yao C Wang J Wang D Gaining confidence in biological interpretation of the microarray data: the functional consistence of the significant GO categories Bioinformatics 2008 24 265 271 10.1093/bioinformatics/btm558 18006543

[B52] Hermans F Tsiporkova E Merging microarray cell synchronization experiments through curve alignment Bioinformatics 2007 23 e64 70 10.1093/bioinformatics/btl320 17237107

[B53] Martin S Zhang Z Martino A Faulon JL Boolean dynamics of genetic regulatory networks inferred from microarray time series data Bioinformatics 2007 23 866 874 10.1093/bioinformatics/btm021 17267426

[B54] Novikov E Barillot E Software package for automatic microarray image analysis (MAIA) Bioinformatics 2007 23 639 640 10.1093/bioinformatics/btl644 17237062

[B55] Royce TE Rozowsky JS Gerstein MB Assessing the need for sequence-based normalization in tiling microarray experiments Bioinformatics 2007 23 988 997 10.1093/bioinformatics/btm052 17387113

[B56] Schumacher M Binder H Gerds T Assessment of survival prediction models based on microarray data Bioinformatics 2007 23 1768 1774 10.1093/bioinformatics/btm232 17485430

[B57] Berg BHJ van den Thanthiriwatte C Manda P Bridges SM Comparing gene annotation enrichment tools for functional modeling of agricultural microarray data BMC Bioinformatics 2009 10 Suppl 11 S9 10.1186/1471-2105-10-S11-S9 PMC3226198 19811693

[B58] Chavan SS Bauer MA Scutari M Nagarajan R NATbox: A Network Analysis Toolbox in R BMC Bioinformatics 2009 10 Suppl 11 S14 10.1186/1471-2105-10-S11-S14 19811679 PMC3152789

[B59] Perkins AD Langston MA Threshold selection in gene co-expression networks using spectral graph theory techniques BMC Bioinformatics 2009 10 Suppl 11 S4 10.1186/1471-2105-10-S11-S4 19811688 PMC3152776

[B60] Li Z Su Z Wen Z Shi L Chen T Microarray Platform Consistency Is Revealed by Biologically Functional Analysis of Gene Expression Profiles BMC Bioinformatics 2009 10 Suppl 11 S12 10.1186/1471-2105-10-S11-S12 19811677 PMC3226184

[B61] Jankun-Kelly TJ Lindeman AD Bridges SM Exploratory Visual Analysis of Conserved Domains on Multiple Sequence Alignments BMC Bioinformatics 2009 10 Suppl 11 S7 10.1186/1471-2105-10-S11-S7 19811691 PMC3226196

[B62] Xu Z Zhang D Hu J Zhou X Ye X Reichel KL Stewart NR Syrenne RD Yang X Gao P Comparative genome analysis of lignin biosynthesis gene families across the plant kingdom BMC Bioinformatics 2009 10 Suppl 11 S3 10.1186/1471-2105-10-S11-S3 19811687 PMC3226193

[B63] Buza TJ Kumar R Gresham CR Burgess SC McCarthy FM Facilitating Functional Annotation of Chicken Microarray Data BMC Bioinformatics 2009 10 Suppl 11 S2 10.1186/1471-2105-10-S11-S2 19811685 PMC3226191

[B64] Zhou X Su Z Sammons RD Peng Y Tranel PJ Stewart N Jr Yuan JS Novel Software Package for Cross-Platform Transcriptome Analysis (CPTRA) BMC Bioinformatics 2009 10 Suppl 11 S16 10.1186/1471-2105-10-S11-S16 19811681 PMC3226187

[B65] Garcia-Reyero N Adelman IR Martinović D Liu L Denslow ND Site-specific impacts on gene expression and behavior in fathead minnows (Pimephales promelas) exposed in situ to streams adjacent to sewage treatment plants BMC Bioinformatics 2009 10 Suppl 11 S11 10.1186/1471-2105-10-S11-S11 19811676 PMC2759673

[B66] Chen B Johnson M Protein Local 3D Structure Prediction by Super Granule Support Vector Machines (Super GSVM) BMC Bioinformatics 2009 10 Suppl 11 S15 10.1186/1471-2105-10-S1-S15 19811680 PMC3226186

[B67] Bright L Swiderski C Burgess S Chowdhary B McCarthy FM Structural and Functional-annotation of an Equine Whole Genome Oligoarray BMC Bioinformatics 2009 10 Suppl 11 S8 10.1186/1471-2105-10-S11-S8 19811692 PMC3226197

[B68] Zollanvari A Cunningham MJ Braga-Neto U Dougherty ER Analysis and Modeling of Time-Course Gene-Expression Profiles from Nanomaterial-Exposed Primary Human Epidermal Keratinocytes BMC Bioinformatics 2009 10 Suppl 11 S10 10.1186/1471-2105-10-S11-S10 19811675 PMC3226183

[B69] Malone BM Perkins AD Bridges SM Integrating phenotype and gene expression data for predicting gene function BMC Bioinformatics 2009 10 Suppl 11 S21 10.1186/1471-2105-10-S11-S20 PMC3226192 19811686

